# Low-Temperature Solution-Processed Gate Dielectrics for High-Performance Organic Thin Film Transistors

**DOI:** 10.3390/ma8105352

**Published:** 2015-10-12

**Authors:** Jaekyun Kim, Chang Jun Park, Gyeongmin Yi, Myung-Seok Choi, Sung Kyu Park

**Affiliations:** 1 Department of Applied Materials Engineering, Hanbat National University, Daejeon 305-719, Korea; jaekyun.kim@gmail.com; 2 School of Electrical and Electronics Engineering, Chung-Ang University, Seoul 156-756, Korea; wanfe89@gmail.com (C.J.P.); rudals219@naver.com (G.Y.); 3 Department of Materials Chemistry and Engineering, Konkuk University, Seoul 143-701, Korea

**Keywords:** organic thin film transistor, gate dielectric layer, self-assembled monolayer, photochemical activation, low-temperature sol-gel method, low-voltage operation

## Abstract

A low-temperature solution-processed high-*k* gate dielectric layer for use in a high-performance solution-processed semiconducting polymer organic thin-film transistor (OTFT) was demonstrated. Photochemical activation of sol-gel-derived AlO*_x_* films under 150 °C permitted the formation of a dense film with low leakage and relatively high dielectric-permittivity characteristics, which are almost comparable to the results yielded by the conventionally used vacuum deposition and high temperature annealing method. Octadecylphosphonic acid (ODPA) self-assembled monolayer (SAM) treatment of the AlO*_x_* was employed in order to realize high-performance (>0.4 cm^2^/Vs saturation mobility) and low-operation-voltage (<5 V) diketopyrrolopyrrole (DPP)-based OTFTs on an ultra-thin polyimide film (3-μm thick). Thus, low-temperature photochemically-annealed solution-processed AlO*_x_* film with SAM layer is an attractive candidate as a dielectric-layer for use in high-performance organic TFTs operated at low voltages.

## 1. Introduction

Solution-processed organic thin-film transistors (OTFTs) have attracted considerable scientific and industrial interest because of their potential application in low-cost flexible electronics, such as active-matrix displays [1], circuitry [2,3], chemical/biological sensors [4,5] and radio-frequency identification (RFID) cards [6]. While small-molecule-based organic electronic materials have exhibited noteworthy performance enhancement [7,8,9,10], semiconducting polymers have also undergone continuous refinement in terms of device performance in recent decades, particularly as regards carrier mobility and operational stability [11,12,13,14,15]. The significant improvement in the carrier mobility of these electronic devices can primarily be attributed to enhancement of the closely packed lamellae structures along the current paths. Further, this improved carrier mobility constitutes a promising opportunity for the application of organic polymers to entry-level organic electronics. For example, it can be expected that solution-processed semiconducting polymers could possibly replace the conventionally used inorganic vacuum-deposited amorphous silicon (a-Si) in switching devices for flexible electrophoretic displays, which require less stringent performance than other devices. However, it is not clear whether solution-processed semiconducting polymers will eventually become applicable to organic light-emitting diodes (OLEDs), which demand significantly higher carrier mobility than the aforementioned switching devices. Recent advancements in the molecular design and processing conditions of high-performance polymer semiconductors have indicated that these materials could fulfill the requirements of various high-end display and electronic-system applications. In order to take full advantage of these polymers from an industrial perspective, the development of low-cost, high-capacitance, and reliable gate dielectric layers is equally important to the development of the high-performance polymer semiconductors themselves [16,17,18,19,20]. The high capacitance and low leakage currents of dielectric layers permit low-voltage operation of semiconducting polymer devices, leading to low power consumption by organic devices containing these polymers. Compared to organic-based dielectric layers, solution-processed inorganic high-*k* dielectrics satisfy the high-capacitance requirement for solution-processed organic polymers more fully, because of their excellent polarizability and chemical stability [17,21,22,23]. Recently, low-temperature solution-processed AlO*_x_* sol-gel films exhibited the relative low leakage current of about 10^−6^ A/cm^2^ at 1 MV/cm [24,25,26], which is suitable for the operation as a gate dielectric layer. Our recent demonstration of the photochemical activation of a solution-processed AlO*_x_* layer for oxide semiconductor devices clearly indicated that dense metal–oxide–metal network formation and condensation at low temperature are readily realizable via a non-thermal activation method [27,28], which is critical in the case of a flexible substrate. Thus, this photochemical activation enables us to fabricate high-performance DPP-based organic polymer devices on a flexible substrate.

In this article, we employ a hybrid gate dielectric layer composed of a photochemically activated AlO_x_ film covered by an octadecylphosphonic acid (ODPA) self-assembled monolayer (SAM) in a high-performance poly-[2,5-bis(2-decyltetradecyl)pyrrolo[3,4-c]pyrrole-1,9-(2H,5H)-dione-(E)-(1,2-bis(5-(thiophen-2-yl)selenophen-2-yl)ethene] (P-29-DPPDTSE) OTFT operated at low voltage (<5 V). ODPA SAM treatment of the AlO*_x_* dielectric surface is used to modify its surface properties, with close intermolecular interactions between neighboring backbones in the P-29-DPPDTSE polymer being favored. This hybrid stack of organic/inorganic materials exhibits low leakage current density with no abrupt breakdown for an applied bias voltage of up to 10 V. Therefore, we achieved high-performance OTFT on a 3-μm-thick flexible polyimide (PI) film.

## 2. Experimental Procedure

In order to prepare the sol-gel precursor for the high-*k* AlO_x_ gate dielectric film, aluminum nitrate nonahydrate (Al(NO_3_)_3_·9(H_2_O)) (Sigma-Aldrich) was dissolved in 2-methoxyethanol at concentrations of 0.3 and 0.8 M, so as to yield solution-processed dielectric films with different thicknesses. Immediately after the precursors were dissolved in the solvent, the resultant solutions were vigorously stirred and kept at 70 °C for more than 12 h to enhance the hydrolysis. For AlO*_x_* film deposition, the solutions were spin-coated at 2000 rpm for 40 s onto sputtered Cr glass or a thin PI/glass substrate with a Cr gate electrode structure to yield metal–insulator–metal (M-I-M) or OTFT device structures, respectively. Hence, thin dielectric layers were formed. Then, the as-deposited samples were photochemically annealed using deep ultraviolet (DUV) annealing for 2 h under a continuous nitrogen flow at room temperature, so as to facilitate condensation and densification of the precursors and formation of the metal–oxide–metal bonding network. During the annealing process, it was observed that the sample temperature gradually increased up to 150 °C as a consequence of the direct thermal radiation, which was generated by a low-pressure mercury UV lamp. Au electrodes for the bottom-contacted devices were then patterned, and the surfaces of the AlO*_x_* dielectric layers were modified by immersing the substrates in the ODPA solution for more than 12 h. This caused the high-performance polymers to favor intermolecular interactions during the solution processing. Finally, P-29-DPPTDTSE polymer films (0.2-wt % chloroform) were prepared through the spin-casting of this polymer onto the substrates at 2000 rpm for 40 s. The resultant films were then annealed at 200 °C for 10 min in the nitrogen-filled glove box. The film thicknesses and surface morphologies of photochemically-annealed AlO*_x_* gate dielectric layers were examined by using the profilometer (KLA-Tencor) and non-contact mode atomic force microscopy (AFM) system (PSIA), respectively. For the electrical characterization of the aforementioned M-I-M and OTFT device structures, capacitance *vs.* frequency and leakage current density *vs.* applied bias behaviors were analyzed using an Agilent LCR meter 4284A and Agilent 4156C analyzer, respectively. The characteristics of the high-performance OTFTs were also examined using an Agilent 4156C semiconductor parameter analyzer. All the electrical measurements were conducted in air and in a dark environment. 

## 3. Results and Discussion

**Figure 1:**
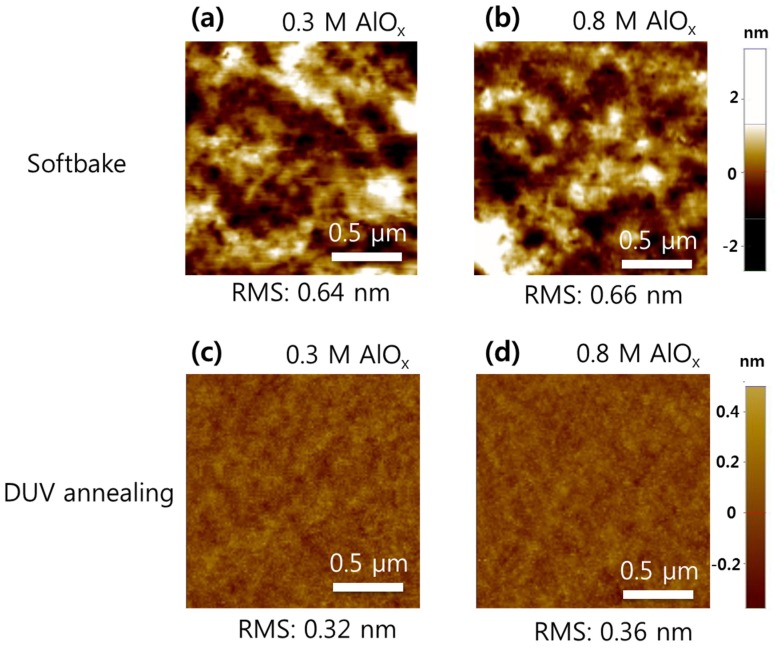
AFM surface scans of (**a**,**b**) softbaked and (**c**,**d**) DUV-annealed AlO_x_ dielectric layers formed with 0.3- and 0.8-M AlO*_x_* sol-gel concentrations, respectively.

Among the various high-*k* metal oxides, AlO*_x_* was chosen for consideration in this study because of its relatively high *k* and wide bandgap, which correspond to a low turn-on voltage and low leakage current. Other high-*k* materials, such as ZrO*_x_* and YO*_x_*, could suffer from a relatively large leakage current due to their low conduction band offsets, although they offer a higher *k* value than AlO*_x_* [29,30,31]. Figure 1 displays the surface morphologies of the photochemically annealed AlO*_x_* dielectric layers before and after DUV annealing, for the different AlO*_x_* sol-gel concentrations of 0.3 and 0.8 M (hereafter referred to as 0.3- and 0.8-M AlO*_x_*, respectively). These two concentrations of AlO*_x_* represent the thin and thick film for use of gate dielectric layers, respectively. AFM analysis revealed that the as-deposited AlO*_x_* films exhibited a substantial decrease in RMS roughness after photochemical activation. Specifically, the initial RMS roughness values of 0.64 and 0.66 nm for the softbaked 0.3- and 0.8-M AlO*_x_* samples were reduced to 0.32 and 0.36 nm, respectively, following DUV annealing. Note that no obvious difference in AlO*_x_* surface roughness for the different sol-gel concentrations was found in this study. This can primarily be attributed to the densification and condensation of the AlO*_x_* precursors upon DUV irradiation, accompanied by the removal of organic species and solvents [24,25]. As a rough dielectric surface often leads to the deterioration of charge carrier mobility in semiconductors due to surface scattering, it is extremely important to keep the surface roughness of the dielectric layer as low as possible [18]. Additional ODPA organic molecules have been known to be effective in suppressing the leakage current of dielectric layer while enhancing the performance of OTFTs [22,32]. It should be also noted that no noticeable difference of root mean square (RMS) roughness was found from 0.3 and 0.8 M concentration of AlO*_x_* precursors. The final thicknesses of the 0.3- and 0.8-M AlO*_x_* dielectric layers were estimated to be approximately 13.5 and 48.2 nm, respectively (not shown). Additional ODPA organic molecules have been known to effectively suppress the leakage current of a dielectric layer while enhancing the performance of OTFTs [22,32], and additional SAM treatment appears to increase the total thickness of the dielectric layer by 2.2 nm; this value roughly corresponds to the ODPA molecule length [22,33]. For estimating the length of ODPA molecules, the step height measurement of AlO*_x_* film with and without ODPA treatment using AFM instrument was carried out following the patterning. As the as-deposited 0.8-M AlO*_x_* layer exceeded 180 nm in thickness, this suggests that DUV irradiation effectively causes the formation of dense metal–oxide films. Therefore, photochemical treatment of sol-gel precursors at medium-range temperatures (~150 °C) can also be used to fabricate OTFTs on a flexible substrate.

**Figure 2:**
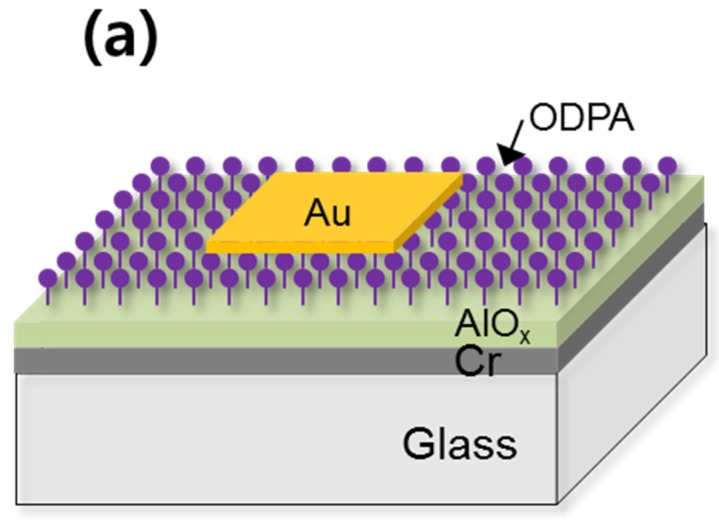

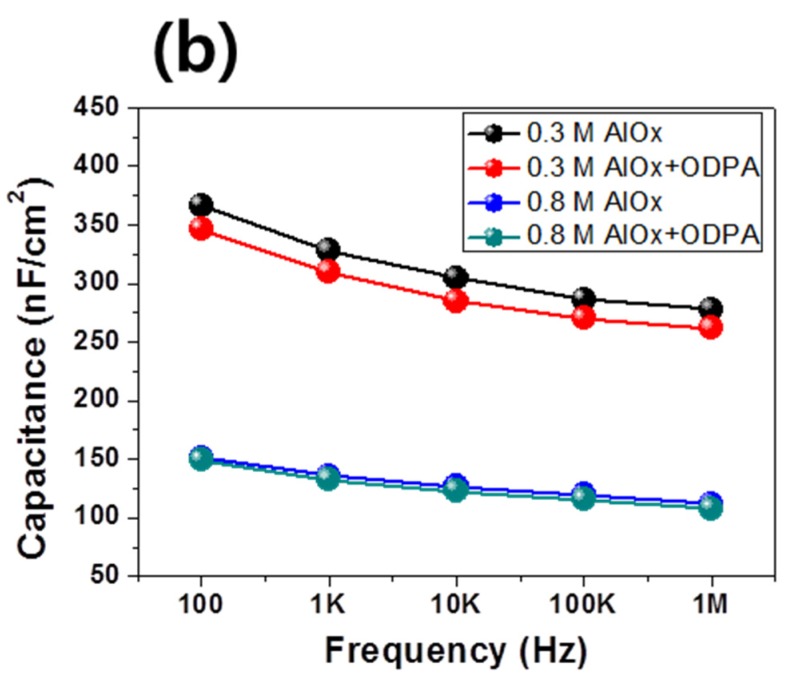
(**a**) Schematic illustration of metal–insulator–metal device structure and (**b**) capacitance *vs.* frequency characteristics of Au-AlO*_x_*/ODPA-Cr/glass devices used in this study.

Areal capacitance measurement of the 0.3- and 0.8-M AlO*_x_* films was employed in order to investigate their performance as gate dielectric layers for low-voltage OTFTs. Figure 2a is a schematic diagram of the M-I-M device structure, while Figure 2b shows the capacitance *vs.* frequency characteristics of AlO*_x_* films with and without ODPA SAM treatment. The capacitance values of the photochemically annealed 0.3- and 0.8-M AlO*_x_* films at 100 Hz were 366 and 149 nF/cm^2^, respectively. The ODPA treatment reduced the capacitance of the 0.3-M AlO*_x_* film slightly, whereas no visible change was apparent in the case of the 0.8-M AlO*_x_*. This difference might be ascribed to the fact that the capacitance of the thicker 0.8-M AlO*_x_* film remains less sensitive to the thin molecule layer. The relatively stable capacitance at higher frequency implies the efficient removal of organic species within the films and also the formation of dense oxide films. Incomplete M-O-M bonding formation and organic residues often yield a noticeable decrease in areal capacitance at high frequency [34,35]. The relative dielectric constant of the photochemically annealed AlO*_x_* was estimated to be approximately 6.3, which is comparable to the values reported in the literature [36].

**Figure 3:**
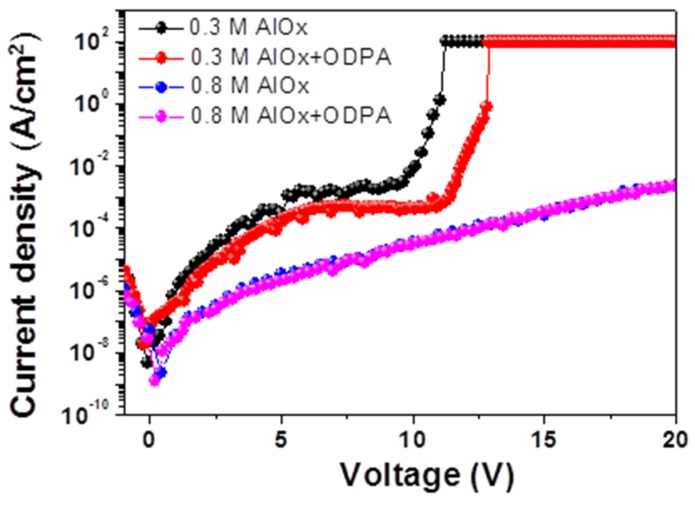
Leakage current characteristics of Au-AlO*_x_*/ODPA-Cr/glass devices with 0.3- and 0.8-M AlO*_x_* sol-gel concentrations.

The leakage current behavior of the photochemically annealed 0.8-M AlO*_x_* film under applied bias exhibited a stable dielectric property, as shown in Figure 3. Notably, no sudden electrical breakdown was observed for currents below 10^−3^ A/cm^2^ at a bias voltage of up to 15 V, which also indicates dense film formation due to the photochemical activation. For lower-operation-voltage OTFTs, a thinner dielectric layer with higher capacitance and an acceptable leakage current is always desired [17,22,37]. As stated previously, the 0.3-M AlO*_x_* yields a 13.5-nm thin high-*k* dielectric layer, thus favoring high capacitance. Here, the 0.3-M AlO*_x_* M-I-M devices seemed to retain a low leakage current up to almost 5-V applied bias, as shown in Figure 3. Therefore, the low-leakage-current behavior of thin AlO*_x_* dielectric films seems to be comparable to the values for high-temperature-annealed oxide gate dielectrics reported in the literature [34,36,38]. It can be understood that low-temperature photochemical activation enables the use of low thermal budget methods for sol-gel-derived oxide insulating layers. This suggests that photochemically annealed thin AlO*_x_* films can be used as high-capacitance dielectric layers to realize OTFTs with low operation voltages. Furthermore, ODPA SAM passivation lowers the leakage current density and increases the breakdown voltage to some extent [22,39].

**Figure 4:**
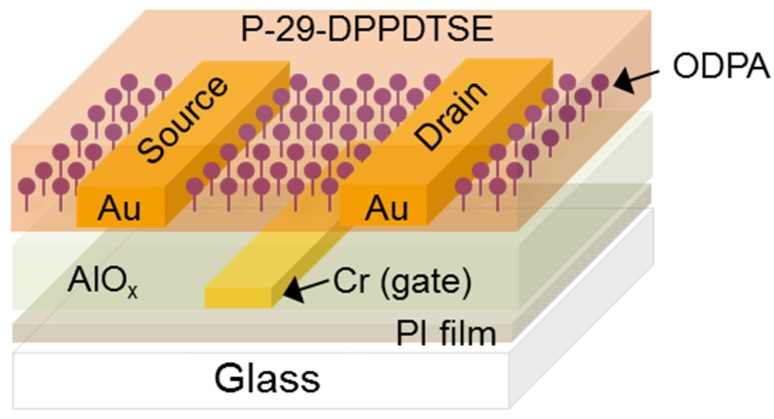
Schematic diagram of high-performance P-29-DPPDTSE polymer semiconductor device on a flexible PI film/glass substrate with DUV-annealed AlO*_x_* gate dielectric layers (not to scale).

**Figure 5:**
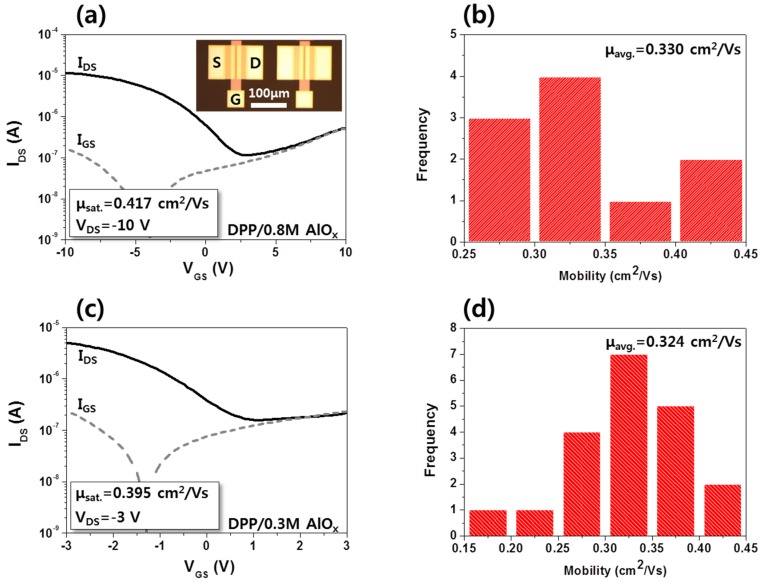
(**a**,**c**) Transfer curves and (**b**,**d**) statistical distributions for saturation mobility of high-performance P-29-DPPDTSE polymer semiconductor devices on flexible PI film/glass substrates with 0.3- and 0.8-M AlO*_x_* sol-gel concentrations, respectively.

Using the fabricated AlO*_x_* films, high-performance P-29-DPPDTSE OTFTs were fabricated on a 3-μm thin PI/glass substrate using a bottom-gate and -contact electrode configuration, as shown in Figure 4. DUV-annealed 0.3- and 0.8-M AlO*_x_* dielectric layers were formed following patterning of the gate electrode. Figure 5a,b show the transfer characteristics and statistical distribution of the saturation mobility of the P-29-DPPDTSE OTFT with a 0.8-M AlO*_x_* gate dielectric layer. The inset of Figure 5a is an optical microscopy image of an OTFT with a 0.8-M AlO*_x_* dielectric layer. Note that no visual defects, such as cracking and inhomogeneity, are apparent. The average mobility of the P-29-DPPDTSE OTFT with a composite dielectrics of ODPA and 0.8 M AlO*_x_* layers was estimated to be as high as 0.330 cm^2^/Vs, while the current on/off ratio (*I*_on_/*I*_off_) appeared to be approximately 2 × 10^2^. Without SAM treatment on AlO*_x_*, we obtained much lower mobility of about 0.03 cm^2^/Vs, possibly due to less ordering in the semiconducting polymer films. The relatively low *I*_on_/*I*_off_ could be due to insufficient isolation of the high-performance organic layer. The flexible substrate made us difficult to physically isolate the high-performance semiconducting layer. A rigid substrate such as silicon allowed us to fabricate OTFTs with 5 × 10^6^ current on/off ratio. A histogram of the mobility distribution of these OTFTs is shown in Figure 5b, indicating the uniformity of the device performance. For the P-29-DPPDTSE OTFT with the 0.3-M AlO*_x_* film, the operation voltage was reduced to 3 V without the appearance of a significant gate leakage current or any device breakdown, as shown in Figure 5c. No significant device performance degradation was observed for this OTFT. In other words, the average mobility was as high as 0.324 cm^2^/Vs. There seems to be no obvious relation between the performance of OTFTs and thickness of dielectric layers. Slight difference of saturation mobility of OTFTs with 0.3- and 0.8-M AlO*_x_* implies that their surface properties appears quite similar, giving rise to similar level of molecular ordering within the semiconducting polymers. A uniform distribution was again obtained, as shown in Figure 5d. It should be noted that both OTFTs on the different flexible films demonstrated high charge carrier mobility of >0.3 cm^2^/Vs on average. Additional optimization of these devices through refinement of both the ODPA treatment and semiconductor processing conditions could further improve the observed device performance.

## 4. Conclusions

In conclusion, it was demonstrated that a high-performance low-operation-voltage P-29-DPPDTSE polymer semiconductor OTFT can be achieved using a photochemically activated AlO*_x_* gate dielectric layer. Additional ODPA SAM treatment enhances the leakage current characteristics of the AlO_x_ film, while also improving the charge carrier mobility performance of the obtained OTFTs. Using a 0.3-M AlO*_x_* film, the fabricated P-29-DPPDTSE OTFT was successfully operated at a gate voltage lower than 3 V and exhibited a saturation mobility of ~0.3 cm^2^/Vs on a flexible PI film substrate. Therefore, the findings of this work indicate that photochemical activation of sol-gel precursors for use in dielectric materials constitutes a promising fabrication technique for solution-processable organic electronics with the capacity for low-voltage operation.

## References

[1] Gelinck G.H., Huitema H.E.A., van Veenendaal E., Cantatore E., Schrijnemakers L., van der Putten J.B.P.H., Geuns T.C.T., Beenhakkers M., Giesbers J.B., Huisman B.H. (2004). Flexible active-matrix displays and shift registers based on solution-processed organic transistors. Nat. Mater..

[2] Klauk H., Zschieschang U., Pflaum J., Halik M. (2007). Ultralow-power organic complementary circuits. Nature.

[3] Kane M.G., Campi J., Hammond M.S., Cuomo F.P., Greening B., Sheraw C.D., Nichols J.A., Gundlach D.J., Huang J.R., Kuo C.C. (2000). Analog and digital circuits using organic thin-film transistors on polyester substrates. IEEE Electron Device Lett..

[4] Mabeck J.T., Malliaras G.G. (2006). Chemical and biological sensors based on organic thin-film transistors. Anal. Bioanal. Chem..

[5] Liao C., Yan F. (2013). Organic semiconductors in organic thin-film transistor-based chemical and biological sensors. Polym. Rev..

[6] Dodabalapur A. (2006). Organic and polymer transistors for electronics. Mater. Today.

[7] Giri G., Verploegen E., Mannsfeld S.C.B., Atahan-Evrenk S., Kim D.H., Lee S.Y., Becerril A., Aspuru-Guzik A., Toney M.F., Bao Z. (2011). Tuning charge transport in solution-sheared organic semiconductors using lattice strain. Nature.

[8] Minemawari H., Yamada T., Matsui H., Tsutsumi J., Haas S., Chiba R., Kumai R., Hasegawa T. (2011). Inkjet printing of single-crystal films. Nature.

[9] Kim Y.-H., Yoo B., Anthony J.E., Park S.K. (2012). Controlled deposition of a high-performance small-molecule organic single-crystal transistor array by direct ink-jet printing. Adv. Mater..

[10] Yuan Y., Giri G., Ayzner A.L., Zoombelt A.P., Mannsfeld S.C.B., Chen J., Nordlund D., Toney M.F., Huang J., Bao Z. (2014). Ultra-high mobility transparent organic thin film transistors grown by an off-centre spin-coating method. Nat. Commun..

[11] Li Y., Singh S.P., Sonar P. (2010). A high mobility p-type DPP-thieno[3,2-b]thiophene copolymer for organic thin-film transistors. Adv. Mater..

[12] Yan H., Chen Z., Zheng Y., Newman C., Quinn J.R., Dötz F., Kastler M., Facchetti A. (2009). A high-mobility electron-transporting polymer for printed transistors. Nature.

[13] Zhang X., Richter L.J., DeLongchamp D.M., Kline R.J., Hammond M.R., McCulloch I., Heeney M., Ashraf R.S., Smith J.N., Anthopoulos T.D., Toney M.F. (2011). Molecular packing of high-mobility diketo pyrrolo-pyrrole polymer semiconductors with branched alkyl side chains. J. Am. Chem. Soc..

[14] Kang I., Yun H., Chung D.S., Kwon S., Kim Y. (2013). Record high hole mobility in polymer semiconductors via side chain engineering. J. Am. Chem. Soc..

[15] Noriega R., Rivnay J., Vandewal K., Koch F.P.V., Stingelin N., Smith P., Toney M.F., Salleo A. (2013). A general relationship between disorder, aggregation and charge transport in conjugated polymers. Nat. Mater..

[16] Yoon M.-H., Yan H., Facchetti A., Marks T.J. (2005). Low-voltage organic field-effect transistors and inverters enabled by ultrathin cross-linked polymers as gate dielectrics. J. Am. Chem. Soc..

[17] Pal B.N., Dhar B.M., See K.C., Katz H.E. (2009). Solution-deposited sodium beta-alumina gate dielectrics for low-voltage and transparent field-effect transistors. Nat. Mater..

[18] Facchetti A., Yoon M.-H., Marks T.J. (2005). Gate dielectrics for organic field-effect transistors: New opportunities for organic electronics. Adv. Mater..

[19] Cho J.H., Lee J., He Y., Kim B.S., Lodge T.P., Frisbie C.D. (2008). High-capacitance ion gel gate dielectrics with faster polarization response times for organic thin film transistors. Adv. Mater..

[20] Kim J., Jang J., Kim K., Kim H., Kim S.H., Park C.E. (2014). The origin of excellent gate-bias stress stability in organic field-effect transistors employing fluorinated-polymer gate dielectrics. Adv. Mater..

[21] Yoon W.-J., Berger P.R. (2010). Atomic layer deposited HfO_2_ gate dielectrics for low-voltage operating, high-performance poly-(3-hexythiophene) organic thin-film transistors. Org. Electron..

[22] Park Y.M., Daniel J., Heeney M., Salleo A. (2011). Room-temperature fabrication of ultrathin oxide gate dielectrics for low-voltage operation of organic field-effect transistors. Adv. Mater..

[23] Beaulieu M.R., Baral J.K., Hendricks N.R., Tang Y., Briseño A.L., Watkins J.J. (2013). Solution processable high dielectric constant nanocomposites based on ZrO_2_ nanoparticles for flexible organic transistors. ACS Appl. Mater. Interfaces.

[24] Wang H., Sun T., Xu W., Xie F., Ye L., Xiao Y., Wang Y., Chen J., Xu J. (2014). Low-temperature facile solution-processed gate dielectric for combustion derived oxide thin film transistors. RSC Adv..

[25] Braquinho R., Salgueiro D., Santos L., Braquinha P., Pereira L., Martins R., Fortunato E. (2014). Aqueous combustion synthesis of aluminum oxide thin films and application as gate dielectric in GZTO solution-based TFTs. ACS Appl. Mater. Interfaces.

[26] Xu W., Wang H., Xie F., Chen J., Cao H., Xu J.-B. (2015). Facile and environmentally friendly solution-processed aluminum oxide dielectric for low-temperature, high-performance oxide thin-film transistors. ACS Appl. Mater. Interfaces.

[27] Jo J.-W., Kim J., Kim K.-T., Kang J.-G., Kim M.-G., Kim K.-H., Ko H., Kim Y.-H., Park S.K. (2015). Highly stable and imperceptible electronics utilizing photoactivated heterogeneous sol-gel metal-oxide dielectrics and semiconductors. Adv. Mater..

[28] Park S., Kim K.-H., Jo J.-W., Sung S., Kim K.-T., Lee W.-J., Kim J., Kim H.J., Yi G.-R., Kim Y.-H., Yoon M.-H., Park S.K. (2015). In-depth studies on rapid photochemical activation of various sol-gel metal oxide films for flexible transparent electronics. Adv. Funct. Mater..

[29] Waggoner T., Triska J., Hoshino K., Wager J.F., Conley J.F. (2011). Zirconium oxide-aluminum oxide nanolaminate gate dielectrics for amorphous oxide semiconductor thin-film transistors. J. Vac. Sci. Technol. B: Microelectron. Nanometer Struct..

[30] Yang W., Song K., Jung Y., Jeong S., Moon J. (2013). Solution-deposited Zr-doped AlO*_x_* gate dielectrics enabling high-performance flexible transparent thin film transistors. J. Mater. Chem. C.

[31] Song K., Yang W., Jung Y., Jeong S., Moon J. (2012). A solution-processed yttrium oxide gate insulator for high-performance all-solution-processed fully transparent thin film transistors. J. Mater. Chem..

[32] Park Y.M., Desai A., Salleo A., Jimison L. (2013). Solution-processable zirconium oxide gate dielectrics for flexible organic field effect transistors operated at low voltages. Chem. Mater..

[33] Ma H., Acton O., Hutchins D.O., Cernetic N., Jen A.K.-Y. (2012). Multifunctional phosphonic acid self-assembled monolayers on metal oxides as dielectrics, interface modification layers and semiconductors for low-voltage high-performance organic field-effect transistors. Phys. Chem. Chem. Phys..

[34] Xu W., Wang H., Ye L., Xu J. (2014). The role of solution-processed high-κ gate dielectrics in electrical performance of oxide thin-film transistors. J. Mater. Chem. C.

[35] Park J.H., Kim K., Yoo Y.B., Park S.Y., Lim K.-H., Lee K.H., Baik H.K., Kim Y.S. (2013). Water adsorption effects of nitrate ion coordinated Al2O3 dielectric for high performance metal-oxide thin-film transistor. J. Mater. Chem. C.

[36] Avis C., Jang J. (2011). High-performance solution processed oxide TFT with aluminum oxide gate dielectric fabricated by a sol–gel method. J. Mater. Chem..

[37] Xu X., Cui Q., Jin Y., Guo X. (2012). Low-voltage zinc oxide thin-film transistors with solution-processed channel and dielectric layers below 150 °C. Appl. Phys. Lett..

[38] Meyers S.T., Anderson J.T., Hong D., Hung C.M., Wager J.F., Keszler D.A., State O., Uni V., Hall G., Cor V. (2007). Solution-processed aluminum oxide phosphate thin-film dielectrics. Chem. Mater..

[39] Xia G., Wang S., Zhao X., Zhou L. (2013). High-performance low-voltage organic transistor memories with room-temperature solution-processed hybrid nanolayer dielectrics. J. Mater. Chem. C.

